# Feasibility of spinal cord imaging at 7 T using rosette trajectory with magnetization transfer preparation and compressed sensing

**DOI:** 10.1038/s41598-023-35853-7

**Published:** 2023-05-31

**Authors:** Sultan Z. Mahmud, Thomas S. Denney, Adil Bashir

**Affiliations:** 1 Department of Electrical and Computer Engineering, Auburn University, Auburn, AL USA; 2 Auburn University MRI Research Center, Auburn University, Auburn, AL USA

**Keywords:** Magnetic resonance imaging, Spinal cord

## Abstract

MRI is a valuable diagnostic tool to investigate spinal cord (SC) pathology. SC MRI can benefit from the increased signal-to-noise ratio (SNR) and contrast-to-noise ratio (CNR) at ultra-high fields such as 7 T. However, SC MRI acquisitions with routine Cartesian readouts are prone to image artifacts caused by physiological motion. MRI acquisition techniques with non-Cartesian readouts such as rosette can help reduce motion artifacts. The purpose of this study was to demonstrate the feasibility of high-resolution SC imaging using rosette trajectory with magnetization transfer preparation (MT-prep) and compressed sensing (CS) at 7 T. Five healthy volunteers participated in the study. Images acquired with rosette readouts demonstrated reduced motion artifacts compared to the standard Cartesian readouts. The combination of multi-echo rosette-readout images improved the CNR by approximately 50% between the gray matter (GM) and white matter (WM) compared to single-echo images. MT-prep images showed excellent contrast between the GM and WM with magnetization transfer ratio (MTR) and cerebrospinal fluid normalized MT signal (MTCSF) = 0.12 ± 0.017 and 0.74 ± 0.013, respectively, for the GM; and 0.18 ± 0.011 and 0.58 ± 0.009, respectively, for the WM. Under-sampled acquisition using rosette readout with CS reconstruction demonstrated up to 6 times faster scans with comparable image quality as the fully-sampled acquisition.

## Introduction

Spinal cord (SC) injury causes sensory and autonomic dysfunctions, and degeneration is linked with several diseases^1–5^. MRI is routinely used for non-invasive diagnosis of SC pathology. However, the ability to detect early or subtle pathological features in SC pathology is limited by spatial resolution, contrast-to-noise ratio (CNR), motion, and physiological noise^6–8^. High spatial resolution is essential for SC MRI to minimize the partial volume effect between the gray matter (GM) and the white matter (WM) due to the small diameter of the cord (~ 1 cm)^7^. Ultra-high field (UHF) MRI can potentially improve SC diagnosis using the increased signal-to-noise ratio (SNR), improved susceptibility contrast, and high spatial resolution. In the last few years, several studies have demonstrated the advantage of UHF MRI of the SC^9–12^. However, routinely used MRI acquisition techniques with Cartesian readouts for SC imaging are susceptible to image artifacts caused by physiological motion^13^. This results in spatial blurring, distortion, loss of contrast, and decreased SNR^6,7^. MRI acquisition techniques with non-Cartesian readouts such as spiral and rosette are inherently insensitive to bulk motion, and can greatly help reduce the motion-related artifacts in SC MRI. The advantages of acquisitions with non-Cartesian readouts have been demonstrated for diffusion MRI, cardiac imaging, fMRI, and real-time imaging^14–17^. Despite the advantages of non-Cartesian readouts, their feasibility for SC imaging has not been fully explored. To our knowledge, only one recent study demonstrated the application of spiral readout for SC MRI at 1.5 T^18^. UHF (7 T and above) MRI offers significant SNR benefits for SC imaging but the potential of acquisition with non-Cartesian readout for high-resolution imaging of the SC at 7 T has not been evaluated. In this work, we demonstrate high-resolution imaging of the SC with rosette readout at 7 T. Rosette readout trajectory offers several advantages over other non-Cartesian trajectories such as flexible trajectory design, smoothly varying gradient waveforms, and self-derived correction of magnetic field inhomogeneity^19–21^.

In addition to standard anatomical images, magnetization transfer prepared (MT-prep) acquisition can enhance the contrast between the GM and the WM and can provide further insight into the SC WM pathophysiology^22,23^. We incorporated an MT-prep module into the pulse sequence to take advantage of short TE and fast trajectory design of rosette readout. Furthermore, shorter acquisition time is beneficial for patient comfort in routine clinical studies. Compressed sensing (CS) has become a valuable tool to accelerate imaging time by exploiting the sparsity in MR images^24–26^. A number of MR applications such as dynamic contrast-enhanced MRI (DCE-MRI), MR spectroscopy, pediatric MRI, phase-contrast MRI for cardiac imaging, and multispectral imaging of the spine have demonstrated the advantages of CS^27–32^. Non-Cartesian trajectories such as rosette are inherently better suited for CS reconstruction^33^.

The goals of this study were to (1) develop a technique for high-resolution SC imaging using rosette readout trajectory at 7 T, (2) demonstrate the feasibility of MT-prep imaging of the SC with rosette readout, and (3) evaluate the application of CS to enable high-resolution imaging of the SC in clinically feasible acquisition time.

## Methods

### Rosette trajectory design

The rosette trajectories oscillate in the radial direction about the origin of k-space with angular frequency ${\omega }_{1}=2\pi {f}_{1}$, simultaneously rotating in the *k*_*x*_*–k*_*y*_ plane with angular oscillation frequency ${\omega }_{2}=2\pi {f}_{2}$. The k-space trajectory is given by^19–21^

1 $$k\left(t\right)={k}_{max}\mathrm{sin}({\omega }_{1}t){e}^{i{\omega }_{2}t}$$

where *k*_*max*_ = *N*_*x*_/(2.*FOV*) is the highest spatial frequency sampled, *N*_*x*_ is the matrix size and *FOV* is the field of view. The values of *f*_1_ and* f*_2_ can be chosen based on the intended shape of the k-space trajectory and the maximum gradient and slew rate allowed by the scanner. We chose *f*_1_ = *f*_2_ for constant maximum gradient strength throughout the readout, which results in a circular trajectory for a single-shot^21^. The corresponding gradient was calculated using $G\left(t\right)=\frac{2\pi }{\gamma }\frac{dk(t)}{dt}$, where $\gamma$ is the ^1^H gyromagnetic ratio^19–21^. The total number of shots to fill up the k-space based on the intended image resolution was^21^

2 $${N}_{sh}=\frac{\pi {N}_{x}}{2}$$

**Figure 1:**
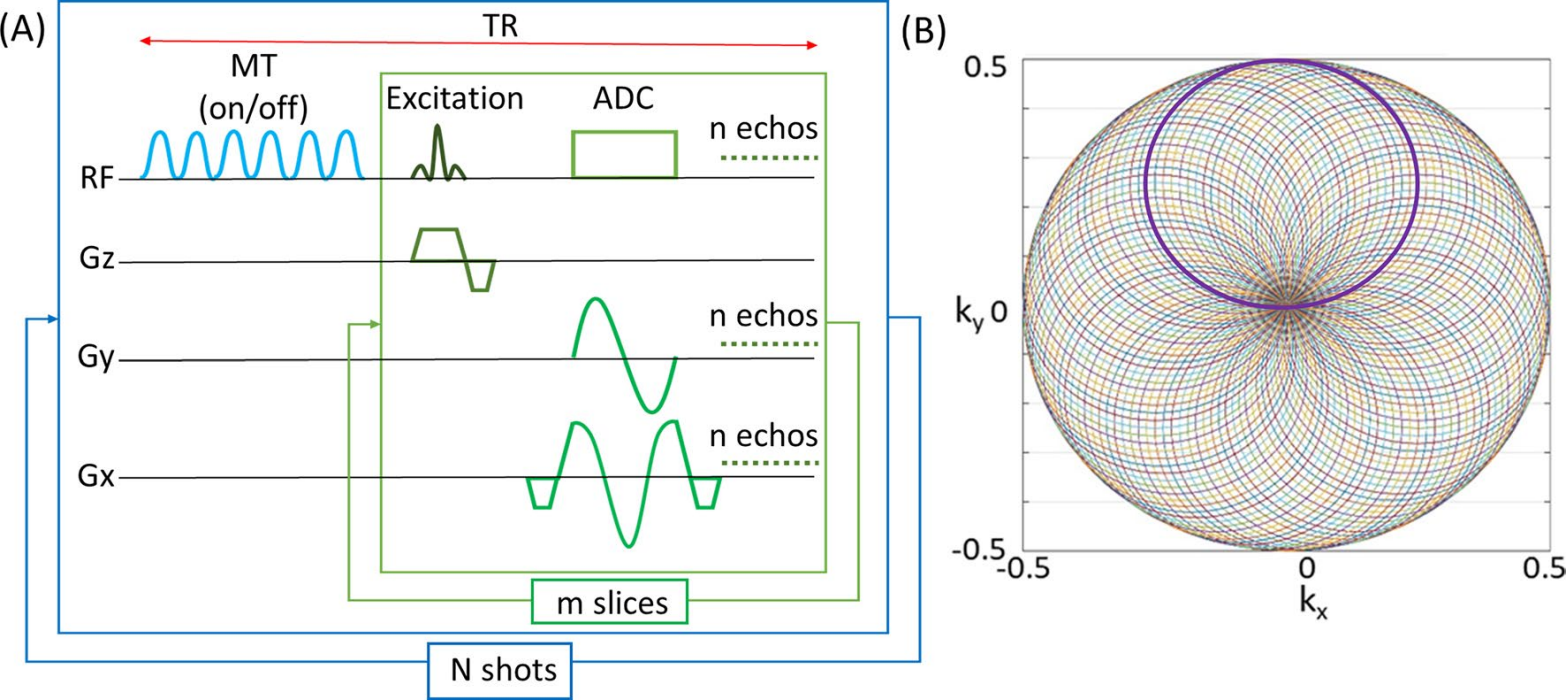
Schematic of the pulse sequence used for imaging with rosette readout trajectory (**A**). The outer box represents the module for one rosette shot and the inner box represents the module for a single-slice. The sequence is composed of 6 MT pulses which can be switched on/off for acquiring rosette data with/without the saturation of the macromolecules, followed by an excitation RF pulse for each slice to acquire *n* echos. *Gy* and *Gx* represent the rosette gradients along the phase encode and the readout axes, and *Gz* is the slice selective gradient. *Gy* starts from 0 and returns to 0 for each acquisition, while *Gx* starts from the maximum value and ends at the same maximum value. The ramp gradients are only required at the beginning and end of the *Gx* gradient. The inner module was repeated to acquire *m* slices and all echoes for each shot. The outer module was repeated to acquire *N* rosette shots. The corresponding rosette trajectory for a single-shot (bold circle) and a total of 603 shots (using Eq. 2) with *f*_1_ = *f*_2_ = 1500 Hz, *FOV* = 192 mm, and matrix size = 384 (**B**).

An MT-prep module was also incorporated into the pulse sequence to generate MT contrast. The schematic of the pulse sequence is shown in Fig. 1A and the rosette k-space trajectory is shown in Fig. 1B.

### Compressed sensing

CS methods use irregular under-sampling schemes to create incoherent aliasing artifacts and nonlinear reconstruction to enforce sparsity in s suitable transform domain, exploiting spatial and temporal correlations to accelerate MRI. Essentially, this is an optimization problem (*l*_1_ regularization) that minimizes this form^24^

3 $$\underset{x}{\mathrm{min}}({\parallel Y-Fx\parallel }_{2}^{2}+\lambda {\parallel \psi x\parallel }_{1})$$

where *Y* is the actual k-space data, *x* is the reconstructed image, *F* is the Fourier transform, $\psi$ is a transform such that $\psi x$ becomes sparse, and $\lambda$ is a regularization parameter weighting the relative importance of the two terms. The symbols ${\parallel \dots \parallel }_{1}$ and ${\parallel \dots \parallel }_{2}^{2}$ represent summations of absolute values and their squares respectively. Equation (3) can be solved using nonlinear conjugate gradient descent algorithm^24^. CS reconstruction parameters similar to the GRASP technique^34^ were used to generate images from a reduced number of rosette shots and compared to the images reconstructed from the fully sampled data.

### Recruitment

The study was approved by the Auburn University Institutional Review Board (IRB). All experiments were performed in accordance with the IRB guidelines and regulations. All the subjects provided informed consent prior to participation in the study. Five healthy subjects (age = 41 ± 13 years, weight = 73 ± 11 kg) participated in the study.

### Pulse sequence

All the experiments were performed on a Siemens 7 T Magnetom (Erlangen, Germany) using 8 channel cervical spine coil. The peak gradient and slew rate of the scanner were 70 mT/m and 200 mT/m/ms, respectively.

The pulse sequence used for imaging with rosette readout trajectory is shown in Fig. 1A. 6 MT pulses, each with a duration of 16.64 ms (bandwidth = 80 Hz), were tuned on/off before the excitation pulse to acquire images with/without MT weighting. MT pulse offset frequency was 500 Hz with a flip angle of 500° to saturate the macromolecules. The MT pulse train (6 MT pulses) was applied once for each TR, where the TR included the acquisition time for all the echoes in all the slices for each rosette shot. Then it was repeated for all the rosette shots. Following the MT pulse train, a 1 ms RF pulse was used for excitation.

Acquisition parameters for imaging with rosette readout were: FOV = 192 mm, matrix = 384, in-plane resolution = 0.5 × 0.5 mm^2^, slice thickness = 4 mm, number of slices = 7, f_1_ = f_2_ = 1500 Hz, flip angle = 39°, sampling time = 1 μs, and TR = 500 ms. The total number of rosette shots acquired was 603 (Eq. 2). Multi-echo images with rosette readout were acquired at TE = 3, 7.8, and 15 ms to demonstrate that combined multi-echo images can improve the contrast between tissues^9^. Images with FLASH readout were also acquired with the same FOV, matrix size, in-plane resolution, slice thickness, and number of slices for comparison. Other parameters for the acquisition with FLASH readout were: TE = 3 ms, TR = 40 ms, flip angle = 10°, duration of each FLASH shot = 40 ms, and the total number of FLASH shots = 384. The total acquisition times for multi-echo and multi-slice SC imaging with rosette readouts were ~ 5 min for 603 shots, ~ 1.67 min (100 s) for 201 shots, and ~ 0.83 min (50 s) for 100 shots; while the total acquisition time for single-echo and multi-slice imaging with Cartesian readouts was ~ 1.9 min.

### Data analysis

Data analysis was performed offline using MATLAB (MathWorks, Natick, MA). The discrepancy in the rosette trajectory due to the magnetic field inhomogeneity was corrected using an estimated linear field map from the 1st two echo images acquired with rosette readout^35^. Then the images were reconstructed for each slice using 2D gridding on a two-fold oversampled grid with a Kaiser–Bessel kernel window W = 4^36^ and density compensation was applied^37^. Images were reconstructed from fully sampled data (603 shots) and a reduced number of rosette shots (201 and 100) using CS^34^. Reconstructed images were segmented using the spinal cord processing toolbox (SCT)^38^.

CNR between GM:WM and CSF:WM was calculated using^39^

4 $$CNR=\frac{{S}_{tissue1, mean}-{S}_{tissue2, mean}}{{S}_{BG, SD}}$$

where *S*_*tissue1,mean*_ and *S*_*tissue2,mean*_ are mean signals from the two tissues of interest, *S*_*BG,SD*_ is the standard deviation of the noise (from background/air).

MTR maps were calculated using^40^

5 $$MTR=1-\frac{{S}_{MT}}{{S}_{o}}$$

where *S*_*o*_ and* S*_*MT*_ are images acquired without and with the MT pulses, respectively.

MTCSF was calculated from the images acquired in the presence of MT pulses only (*S*_*MT*_)^41^. CSF was segmented as described above and the mean signal from the CSF region was determined (*S*_*CSF*_). MTCSF maps were then generated by normalizing *S*_*MT*_ images to the mean signal from the CSF region (*S*_*CSF*_)

6 $$MTCSF=\frac{{S}_{MT}}{{S}_{CSF}}$$

All results are reported as mean ± standard deviation.

### Statistical analysis

CNR, MTR, and MTCSF values across all the slices and voxels for each subject were averaged and used for statistical analysis (effective n = 5). Due to the small sample size (n = 5), a two-sided non-parametric test (Wilcoxon rank sum test) was used to compare the CNR from fully sampled (603 shots) single-echo images acquired with rosette readouts, under-sampled (201 and 100 shots) single-echo CS images acquired with rosette readouts, and fully sampled multi-echo averaged images acquired with rosette readouts to the single-echo images acquired with Cartesian readouts. A paired, non-parametric test (Wilcoxon signed rank test) was performed to compare the MTR and MTCSF values between the GM and WM.

## Results

**Figure 2:**
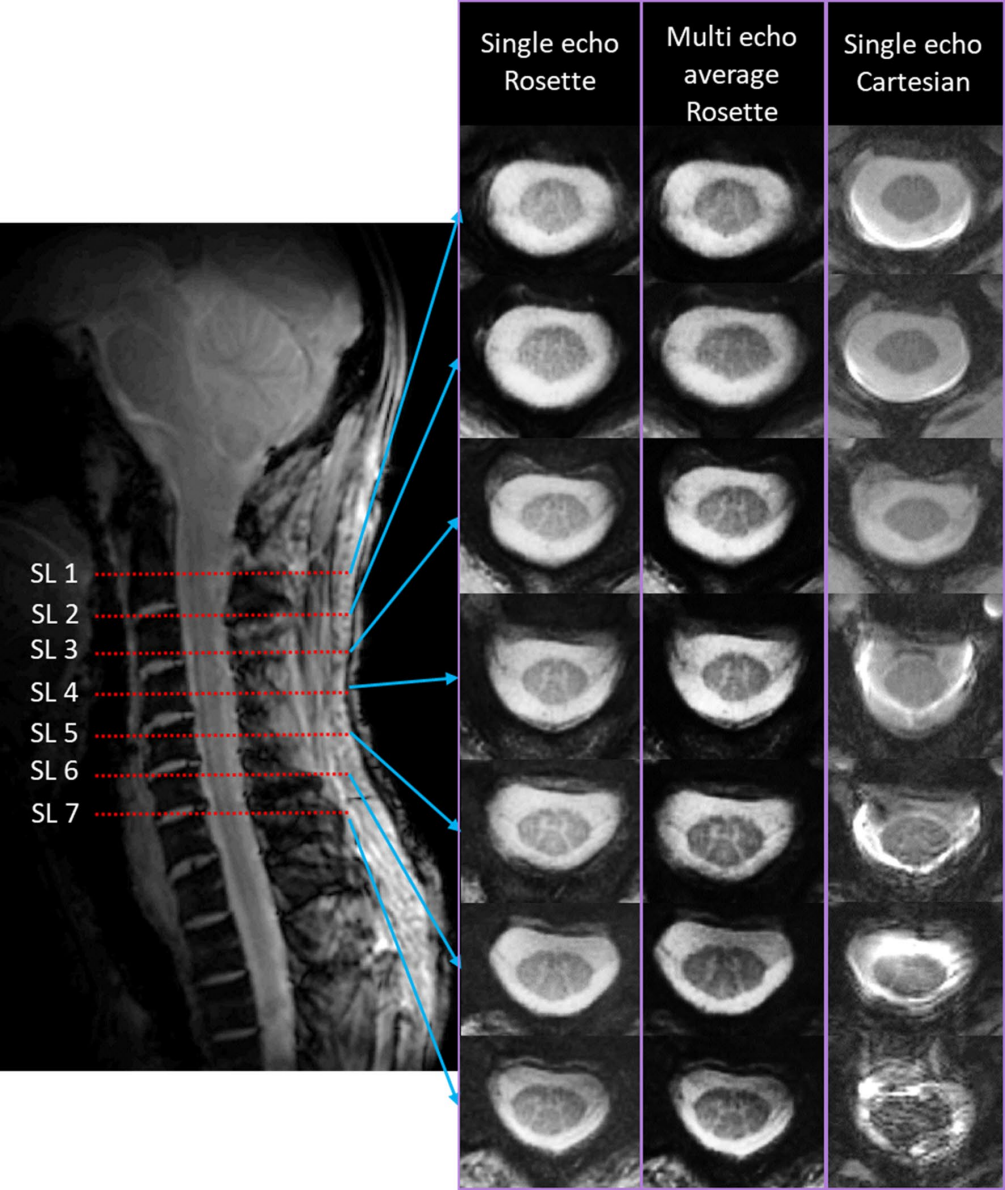
Slice locations on the SC (SL 1–SL 7) and corresponding single-echo (TE = 3 ms; 603 shots) images acquired with rosette readouts, multi-echo average (TE = 3, 7.8 and 15 ms; 603 shots) images acquired with rosette readouts, and single-echo (TE = 3 ms) images acquired with Cartesian (FLASH) readouts from a representative subject. Severe motion artifacts are visible in the images (SL 4–SL 7) acquired with Cartesian readouts, which are barely noticeable in the images acquired with rosette readouts. Multi-echo averaged rosette-readout images show increased contrast between different types of tissues compared to the single-echo rosette-readout images.

Maximum gradient and slew rate were 35.23 mT/m and 105.7 mT/m/ms respectively for the rosette design used in this study. Representative single-echo fully sampled images acquired with rosette readouts (603 shots) from 7 slices of the spine at TE = 3 ms are shown in Fig. 2. Single-echo images acquired with rosette readouts show reduced motion artifacts with sharp contrasts between the GM, the WM, and the CSF, compared to single-echo images acquired with FLASH readouts (TE = 3 ms) (Fig. 2). Slice 4–7 (SL 4–SL 7 in Fig. 2) show severe motion artifacts in the images acquired with FLASH readouts, while the images acquired with rosette readouts do an excellent job of reducing the motion-related blurring. On average, ~ 37% of the images (per subject) acquired with Cartesian readouts demonstrated severe motion artifacts (slice locations comparable to Fig. 2). None of the images acquired with rosette readouts from all the subjects demonstrated any severe motion artifact. Images acquired with rosette readouts demonstrated overall higher CNR than the images acquired with FLASH readouts (Table 1). Averaging multiple echo time images acquired with rosette readouts (TE = 3, 7.8, and 15 ms) significantly improves the contrast among the GM, the WM, and the CSF compared to the single-echo images (TE = 3 ms) (Fig. 2). Multi-echo averaged images acquired with rosette readouts also achieved the maximum CNR (Table 1).

**Table 1:** CNR (GM:WM and CSF:WM) for single-echo images acquired with Cartesian readouts, single-echo fully sampled (603 shots) images acquired with rosette readouts, single-echo under-sampled CS images (201 and 100 shots) acquired with rosette readouts, and multi-echo fully sampled images acquired with rosette readouts from all the subjects.

	CNR (P)	
	GM:WM	CSF:WM
Single-echo Cartesian (FLASH)	1.3 ± 0.3	31.8 ± 5.7
603 shots single-echo rosette	4.8 ± 1.1 (0.02)	59.6 ± 7.2 (0.01)
201 shots-CS single-echo rosette	4.1 ± 1.1 (0.006)	56.8 ± 8.7 (0.03)
100 shots-CS single-echo rosette	2.9 ± 0.9 (0.01)	47.6 ± 9.8 (0.003)
603 shots multi-echo average rosette	7.2 ± 1.7 (0.007)	67.1 ± 6.3 (0.04)

TE = 3 ms for all single-echo data; and TE = 3, 7.8, and 15 ms for all multi-echo data. p values were calculated by comparing the CNR to the single-echo Cartesian-readout images.

**Figure 3:**
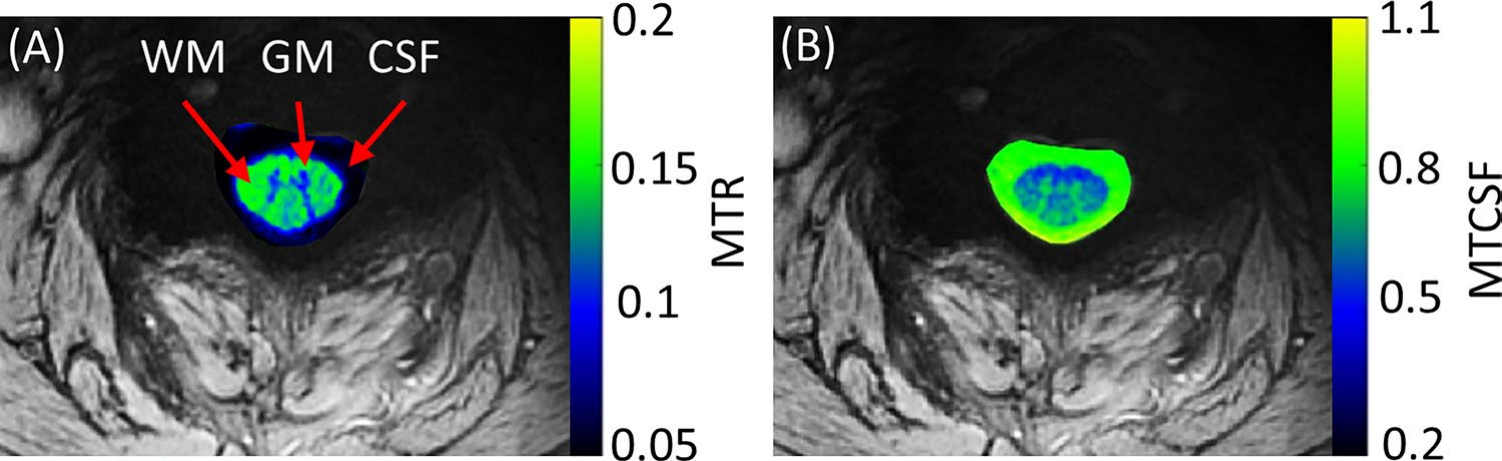
MTR and MTCSF maps (**A**,**B** respectively) from a representative subject. MTR and MTCSF show inverted contrast between different types of tissues.

MTR and MTCSF maps from a representative subject are shown in Fig. 3. MTR was lower in the GM (0.12 ± 0.017) compared to the WM (0.18 ± 0.011) (p < 0.009), averaged across all the subjects. CSF did not show any MTR confirming minimal spillover saturation of the MT pulses^6^. MTCSF (Fig. 3b) produces tissue contrast inverted to that of MTR. The signal was higher for the MTCSF in the GM (0.74 ± 0.013) compared to the WM (0.58 ± 0.009) (p < 0.01), averaged across all the subjects.

**Figure 4:**
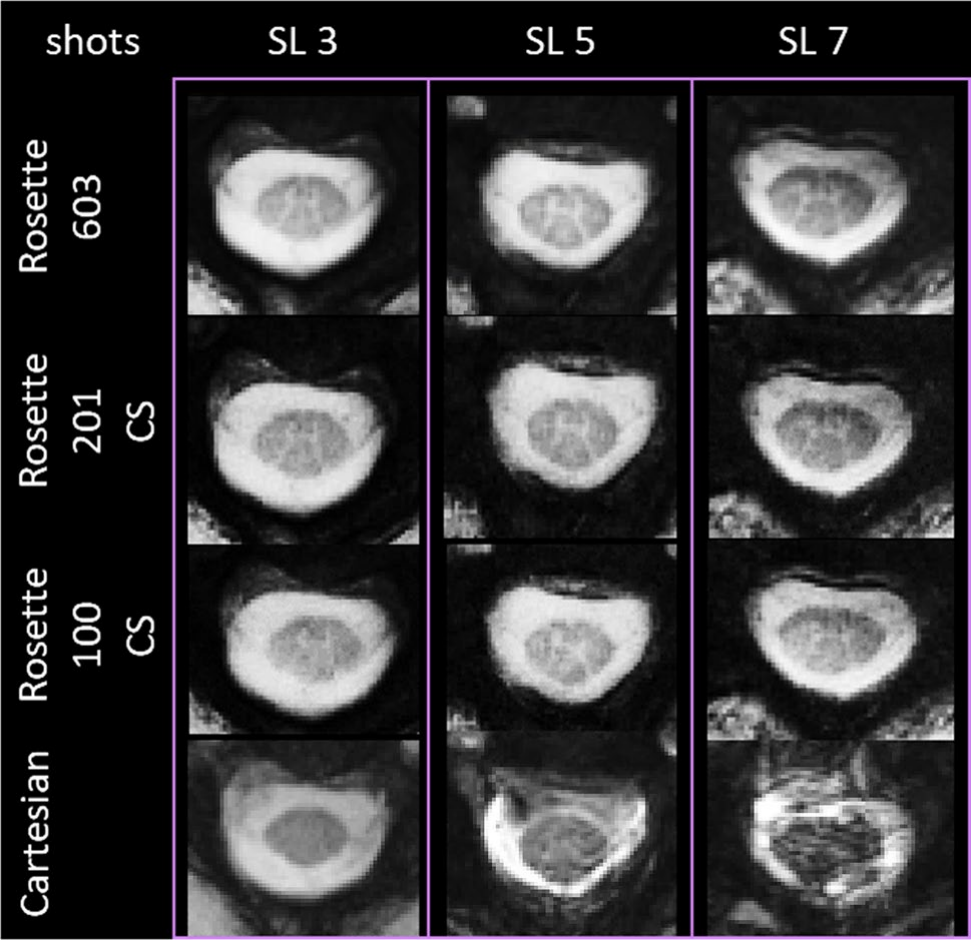
Evaluation of CS for imaging of the SC with rosette readout. Rosette-readout images reconstructed from fully sampled k-space (603 shots) from three representative slice locations (SL 3, SL 5, and SL 7) are shown in the top row. Rosette-readout images reconstructed using CS from under-sampled k-space (201 and 100 shots, 2nd and 3rd row, respectively) show image quality similar to the ones reconstructed from fully sampled k-space and have better image quality than the images acquired with Cartesian (FLASH) readouts (bottom row).

CS reconstruction of under-sampled data [100 (6 × under-sampled) and 201 (3 × under-sampled) shots] shows image quality better than the images acquired with Cartesian readouts and comparable to the images reconstructed from fully sampled data (603 shots) (Fig. 4). CNR from 201 shots-CS images was higher than the images acquired with Cartesian readouts and very comparable to the 603 shots images (Table 1). While the images reconstructed from 100 shots with CS lose some details compared to 603 shots images but visually demonstrate better image quality than the images acquired with FLASH readouts (Fig. 4). CNR from 100 shots-CS images was also higher than the images acquired with Cartesian readouts but lower than 603 shots-CS images (Table 1). Under-sampled acquisition using rosette readout with CS reconstruction allowed us to reduce the acquisition time by up to 6 × and can potentially help with patient comfort.

## Discussion

This study is the first report to demonstrate the high-resolution MRI of the SC using rosette readout trajectory at 7 T. We also demonstrated magnetization-prepared high-resolution MT images and the feasibility of compressed sensing for SC imaging with rosette readout. The advantages of MRI acquisition with rosette readout compared to the acquisition with routine Cartesian readout, including low susceptibility to bulk motion, can greatly benefit the investigation of SC pathology in a clinical setup. MT-prep imaging with rosette readout can provide further insight into WM pathology^22,23,41^. We also showed that CS can significantly accelerate the data acquisition using rosette readout with better image quality and CNR than typical Cartesian readout (Fig. 4; Table 1). MRI acquisition with rosette readout technique has been demonstrated in applications such as MR spectroscopic imaging (MRSI), fMRI, etc.^14,20,21^. This study demonstrates that rosette readout has significant benefits over typical Cartesian readout for SC MRI (Figs. 2, 4; Table 1). This can make it a useful tool for routine high-resolution free-breathing SC imaging in clinical applications.

Despite the obvious potential advantages of UHF MRI of the SC, there are still considerable challenges associated with 7 T SC imaging that need to be overcome. Previous studies on SC imaging at 7 T have mostly concentrated on the design of the RF coil^9,10,12,42,43^. CNR and motion artifacts are some of the biggest challenges that need to be addressed for high-resolution SC imaging. In our study, SC images acquired with rosette readouts showed significant improvement over the images acquired with Cartesian (FLASH) readouts. Very good contrast between different types of tissues was visible in the images acquired with rosette readout (Fig. 2). Averaging multiple echo images further improved the contrast (Fig. 2). Acquisitions with rosette readouts demonstrated an excellent ability to reduce the physiologic motion artifacts compared to the Cartesian readouts, especially towards the lower end of cervical SC, which is more prone to respiratory motion (Fig. 2). None of the images acquired with rosette readout from all the subjects demonstrated any severe motion artifact. Although images acquired with rosette readouts showed better contrast and CNR between different types of tissues compared to the images acquired with Cartesian readouts (Fig. 2; Table 1), different flip angles and TRs used for the acquisitions with rosette and FLASH readouts might be a contributing factor. A more accurate quantitative comparison of CNR between FLASH and rosette can be investigated in a future study by using the exact same acquisition parameters for both acquisitions. The total acquisition time for fully sampled (603 shots) multi-echo and multi-slice imaging with rosette readouts was ~ 5 min, which was higher than the single-echo and multi-slice fully sampled imaging with Cartesian readouts (~ 1.9 min). The rosette sequence included MT pulse train and multi-echo acquisition, which required a longer TR and hence led to a longer acquisition time. The acquisition times for multi-echo and multi-slice under-sampled acquisitions with rosette readouts (~ 1.7 min for 201 shots and ~ 0.8 min for 100 shots, respectively) were lower than the acquisition time with Cartesian readouts. Additionally, the peak gradient and slew rate (35.23 mT/m and 105.7 mT/m/ms, respectively) used in this study for the rosette trajectory were approximately half of the maximum capacity of the 7 T scanner (70 mT/m and 200 mT/m/ms, respectively). The rosette sequence parameters can be further optimized to achieve faster acquisition by using a higher value for *f*_1_ and *f*_2_ (Eq. 1).

In addition to standard anatomical images, MT can provide valuable pathological information in diseases such as multiple sclerosis (MS), adrenomyeloneuropathy (AMN) disorder, and other neurological disorder^23,41,44,45^. MTR depends on the tissue macromolecular concentration. MTR is usually higher in the WM due to the high concentration of macromolecules like protein and lipids present in the myelin sheath. On the other hand, as per the definition of MTCSF, the tissue contrast in MTCSF is opposite to that of MTR (i.e. higher in the GM and lower in the WM). WM pathology such as demyelination causes the macromolecular concentration in the WM to drop and consequently affects the MTR and MTCSF and they can be valuable non-invasive biomarkers in WM diseases^22,23,41^. Our study demonstrated good MT contrasts between tissues (Fig. 3), however, the average MTR in this study was a little low (0.12 ± 0.017 and 0.18 ± 0.011 in the GM and the WM, respectively). This is because the MT saturation pulse power was kept low to reduce the specific absorption rate (SAR) for in vivo imaging at 7 T. MT contrast can be improved by optimization of MT RF pulses, offset frequency, and increasing the number of rosette shots for each MT-prep pulse. The optimization of these parameters was not explored in this study. The spillover effect of the MT pulses at an offset frequency of 500 Hz was found to be < 2% in the head^46^.

CS reconstruction of the SC images acquired with rosette readout demonstrates that the k-space can be significantly under-sampled without severely compromising the image quality. The optimum number of shots for the intended resolution in this study was 603 (Eq. 2). Reconstructed images and CNR from reduced 201 shots are better than the images acquired with typical Cartesian readout and comparable to the images reconstructed from 603 shots (Fig. 4; Table 1), resulting in a three-fold acceleration in acquisition time. Images and CNR from 100 shots (six-fold acceleration in imaging time) show a downgrade from 603 shots images, however, they still show better quality and fewer motion artifacts than the images acquired with typical Cartesian readout (Fig. 4; Table 1). We used CS reconstruction parameters similar to the GRASP technique, which uses total variation (TV) for the sparse representation of MR images ($\psi$ in Eq. 3)^34^. Although this technique provided good performance for the SC imaging in this study up to sixfold acceleration, other sparsifying transforms such as wavelet transform and optimization of $\lambda$ (Eq. 3) can potentially improve the performance of the CS^24^.

In summary, we demonstrated the feasibility of high-resolution imaging of the SC using rosette readout trajectory at 7 T, with the addition of MT-weighted imaging and the application of CS. This technique can be very useful to reduce motion artifacts compared to imaging techniques with routine Cartesian readout. Additionally, MT-weighted imaging and CS can help diagnose WM pathology and reduce imaging time, which can be beneficial for clinical applications.

## Data Availability

The datasets used and/or analyzed during the current study are available from the corresponding author on reasonable request.
